# Ecosystems of Co-Creation

**DOI:** 10.3389/fsoc.2021.642289

**Published:** 2021-02-26

**Authors:** Jennifer Eckhardt, Christoph Kaletka, Daniel Krüger, Karina Maldonado-Mariscal, Ann Christin Schulz

**Affiliations:** Sozialforschungsstelle Dortmund, Faculty for Social Sciences, TU Dortmund University, Dortmund, Germany

**Keywords:** co-creation, social innovation, design, citizen science, ecosystem

## Abstract

Citizen science is becoming increasingly important as a new and participative mode of knowledge production. An essential element of citizen science is co-creation. Co-creation is by no means limited to a modus operandi for participatory science, but introduces a form of collaborative way of working with society in the sense of citizen science. Results from the H2020 SISCODE project show that co-creation is located inside and between different sectors of society. This article focuses on the question of how co-creation can be better understood in different contexts, and presents a heuristic model that has already been used for case study analyses in the SISCODE project. After an introduction to the field of co-creation and a brief description of the heuristic model, its capability is exemplarily demonstrated *via* application to two selected cases, followed by a discussion of central learnings and implications for further research on co-creation.

## Introduction

In the last decades, there has been an increasing political will in the European Union to democratize innovation processes and to strengthen societal participation in innovation and research. A major reason for this development seems to be the goal to find better solutions for social problems with the participation of all actors affected by these solutions (BEPA, 2010). For this purpose, the concept of responsible research and innovation (RRI) and the idea of mission-oriented research were established (Mazzucato, 2018) and became prominent. The call for more participation of civil society in research and innovation is linked to the rise of citizen science, a concept that refers to the opening of science toward society (Hecker and Wicke, 2019; Ostermann-Miyashita et al., 2019). Tried out by natural sciences with a focus on sustainability, this concept is nowadays shaping practice-oriented research in social science, too (Kullenberg and Kasperowski, 2016; Hecker et al., 2018). Not only science opens up to society but also politics and business involve citizens in producing new knowledge and in developing innovations. The results are increasingly participatory, joint innovation processes produced by various stakeholders with diverse knowledge and stakes and from various contexts. Such joint, participatory innovation processes are described with the concept of co-creation (Leclercq et al., 2016; Hochgerner, 2018). In this respect, co-creation, understood as a participatory multi-stakeholder innovation process, forms the context in which citizen science is realized. However, despite a consensus on the participative, cross-sectoral character of co-creation, comprehensive definitions are still not established in research. Co-creation can be understood as a method, process, or service (Sanders and Stappers, 2008; Brandsen and Honingh, 2018). It can be used in the public sector, society, business, and universities (Voorberg et al., 2014; Jørgensen, 2018). One of the main characteristics of co-creation is the value of collaboration with different stakeholders, the creation of a collaborative platform, and the involvement of stakeholders in different innovation processes (Leclercq et al., 2016; Hochgerner, 2018). Some authors recognize at least three types of co-creation with citizens: co-implementation, codesign and initiation, and processes in which citizens participate in different ways (Voorberg et al., 2014).

In SISCODE (codesign for society in innovation and science), a three-year European Union–funded project, the use of co-creation led by design principles takes center stage. Assuming that the use of design methods and principles plays a crucial role in co-creation and its successful implementation, SISCODE wants to make sense of practices of co-creation by design (“co-design”) in different contexts. From the successful implementations of co-design, conclusions should be drawn for a better exploitation of co-design in the fields of RRI and policy-making. To do so, a theoretical background through an analysis of European cases and real-life experimentations was developed. The research heuristic, used as a lens to examine practices of co-creation and factors influencing their success and failure, is presented in this article. In line, this article argues that success and failure of participatory innovation processes must be understood through different and interlinked factors on distinguishable levels within any given ecosystem. Its specific contribution is the exemplary application and discussion of a social innovation ecosystem heuristic, developed by Kaletka et al., (2017, 85), to the field of co-creation. Furthermore, the discussion also highlights potential for a further development and application of the model, based on the experiences made during its actual application in the process of analysis in the SISCODE project. Therefore, the aim is to answer two questions: (1) What can be learned from the application of the research heuristic from social innovation research to the analysis of co-creation ecosystems in SISCODE? (2) What conclusions can be drawn from this application for future research? This article, hence, contributes to a better understanding of the research object of co-creation. Although co-creation concerns traditional research fields, it is at the same time a separate field of research, not despite its interdisciplinary and transdisciplinary character, but precisely because of this character. While co-creation is a modus operandi of specific participatory activities across fields like policy-making, service, and product development, it is not limited to single domains and cannot be understood with a focus limited to, for example, politics, engineering, or economics. A major starting point for this article is the thesis that co-creation can only be understood from a transdisciplinary perspective, hence, taking into account its context-specificity with a variety of problems addressed by a variety of actors.

## Ecosystems of Co-Creation as an Empirical Field

This section creates an overview of the terms and concepts used in this article with the aim to provide guidance and a joint understanding. As this article seeks to share experiences from studying practices of co-creation in different fields of action and various settings, *Co-Creation and Its Different Contexts in Innovation* elaborates different approaches to co-creation to illustrate its conceptual proximity to the field of social innovation despite their differences. Building on that, *An Open Heuristic to Social Innovation Ecosystems* details this proximity to introduce an open heuristic model, which can serve as a search pattern to describe both social innovation and—with adaptions presented in this paper—co-creative initiatives and practices. Leading over to the case-study examples of its application, *Application to Co-creation Initiatives* briefly explains how the heuristic was adapted for the SISCODE project.

### Co-Creation and Its Different Contexts in Innovation

Co-creation has been a widespread concept implemented in marketing, whereas other fields have recognized its valuable elements of collaboration, value-creation, and as an engagement platform (Leclercq et al., 2016). Research shows that the understanding of co-creation is changing and nowadays it is not only seen as a method but also as a process where different stakeholders are involved in different stages of an innovation (Leclercq et al., 2016; Hochgerner, 2018), or as a part of a system where organizations are involved to make decisions. Some of these perspectives are presented in the following.

Co-creation as a method is used in design as a way to promote participatory practice (Sanders and Stappers, 2008). Design co-creation is also a method in action research, in which workshops with stakeholders are facilitated in formal design (Jones, 2018, 8). Besides these methods, co-creation is also used as design focus on collaborative processes involving different stakeholders to generate ideation to guarantee first-stage participation of all actors affected by a future solution.

Co-creation as a process or service is a perspective that comes from business, which became popular in the public sector (Brandsen and Honingh, 2018, 9). In contrast to Brandsen and Honingh (2018), Voorberg et al., (2014) distinguished between three types of co-creation: citizens as co-implementer (citizens are involved in services implemented by government), citizens as codesigner (citizens are involved in the process of service), and citizens as initiator (citizens take up the initiative). Besides the public sector, co-creation is also concerned at a strategic level—when citizens are involved in initiating the general planning of a service (Brandsen and Honingh, 2018, 13). In this interpretation of co-creation, service is in foreground, whereby its initiation and planning are in the focus.

Regardless of whether co-creation is conceptualized as a method, process, or service, it can be summarized as an intervention that changes the way things are done in several fields. In particular, it addresses changes in traditional cultural and organizational practices from a top-down approach to a bottom-up approach in which citizens or end-users become actors in a development process. The field in which co-creation takes place is a crucial dimension to observe when trying to describe and analyze the modes of action of co-creation and the changes it triggers.

The following explanations seek to shed light on an understanding of co-creation in its contexts, leading to the general notion of co-creation as a partial practice of social innovation processes and participative innovation processes in more general terms. As elaborated, co-creation is a way to collaborate for decision-makers, experts, and other stakeholders in various contexts (Jones, 2018, 14). In large organizations, for example, in the public sector or healthcare system, the collaboration through co-creation activities is used to optimize products or services (ibid). Co-creation promotes a culture of innovation (Sørensen and Torfing, 2015) because it engages stakeholders who are not usually involved. Through this process, different stakeholders do not only collaborate but also experiment. It also allows the development of their skills and opens up a new field for innovation practices, which can be applied in different societal sectors and social services. In the public sector, it refers, for example, to the commitment of citizens in policy-making through the early-stage participation of citizens in the definition and solution of local problems. In business, it refers to providing the “user” with an active and collaborative role at various stages of the process (Maase and Dorst, 2006; Voorberg et al., 2014), what is often used by entrepreneurs and start-ups. Finally, co-creation in academia and science is observed through spaces of exchange between citizens and researchers, whereby citizens participate in the research process (e.g., citizen science) (Voorberg et al., 2014).

An overall perspective of co-creation shows that it pursues a nonlinear logic, which embodies a multi-dynamic and multi-contextual process. It is often described as a bottom-up approach (Kumari et al., 2019) that operates on different levels whereby citizens and other stakeholders are the key actors. Stakeholders with different backgrounds in culture, belief, and knowledge take different roles and integrate them into a co-creation process. To take this into account, the tools, instruments, and methods used within the co-creation process need to be well aligned and suitable for the respective contexts to promote its success.

As recent research indicates, processes of co-creation are frequently driven by design principles, often without any notice or intention from initiators or participants (Rizzo et al., 2018; Smallman and Patel, 2018). The introductorily mentioned project SISCODE is dedicated toward these specific practices of co-creation and delivers insights and evidence to stimulate openness toward co-creation in science, technology, and innovation (STI), policy-making, as well as in responsible research and innovation (RRI). In the project, co-creation is understood as “a bottom-up and design-driven phenomenon that is flourishing across European contexts like FabLabs, Living Labs, Social Innovation, smart cities, communities, and region” (Eckhardt et al., 2020a, 10). The overall aim of the project is the description of various co-creation approaches in different fields and their respective ecosystems to understand social dynamics (Eckhardt et al., 2020a, 11). Once implemented, the cultural and organizational transformation through co-creation can be seen in established practices and power-shifting policies.

These explanations already point to the close relation of co-creation to social innovation, understood as a new configuration of practices with the overall goal to address social problems in a way they were not addressed through established practices before (cf. Howaldt and Schwarz, 2010). As co-creation involves new social practices and new modes of interaction, it can be considered as an emerging and currently diffusing social innovation itself. Furthermore, Terstriep et al., (2020) emphasized that processes of social innovation are often determined by co-creation, because cross-sectoral cooperation and the participation of all actors involved are a success factor for its emergence and fruitful development (cf. Carayannis and Campbell, 2009). Therefore, co-creation can be conceptualized as an important partial practice within the process of (participatory) social innovation processes. In either way, the question rises why some practices gain momentum, become implemented, normalized, and routinized, and some other practices decline and vanish. At this point the latest the totality of contextual factors, influencing the pathway of practices of social innovation and co-creation, for example, cultural and organizational structures, becomes relevant. The next section is dedicated to a deeper description of this ecosystem and lays down a way to openly examine it in empirical research in the field.

### An Open Heuristic to Social Innovation Ecosystems

The concept of ecosystems originally comes from the natural sciences, where it defines a community of organisms and their environment in an interactive and complex system (Willis, 1997). This concept has been transferred across disciplines, including the social sciences, where community capacity has been added as a key element (Donoghue and Sturtevant, 2007).

A review of the literature shows that the concept of the ecosystem provides a framework for understanding and studying the interaction of various actors, institutions, and contexts in society (Kumari et al., 2019). One of the main research questions in the literature is as follows: What are the key dimensions and what are the barriers and drivers of an ecosystem (Bason, 2010, 25)? However, there is a lack of common understanding of the concept, so there are major difficulties in comparing ecosystems (O’Neill et al., 1986; Edquist, 2011). Although there is a gap in the literature with a unified perspective (Terstriep et al., 2020), more recently, some efforts have been made to understand the social, cultural, and institutional aspects of an ecosystem. For example, earlier research shows a focus on the business ecosystem (Anggraeni et al., 2007; Williamson and De Meyer, 2012; Spigel, 2017), while other studies explore innovation ecosystems (Adner, 2006; Adner, 2012) and more recently social innovation ecosystems (Kaletka et al., 2017; Pel et al., 2020; Terstriep et al., 2020). Authors such as De Vasconcelos Gomes et al., (2018) recognize that there is a transition in the theoretical perspectives of business ecosystems to innovation ecosystem. They point out that one of the main differences between business ecosystems and the innovation ecosystem lies in the value of co-creation practices “innovation ecosystem is related to value creation while business ecosystem refers to value capture” (De Vasconcelos Gomes et al., 2018, 31).

Social innovation ecosystems are complex systems of interaction between various stakeholders. Co-creation practices in social innovation ecosystems refer to the agreement between multiple stakeholders (Kumari et al., 2019; Jütting, 2020; Pel et al., 2020) to achieve a common goal. This means that within an ecosystem, there is more capacity generation than as an individual; this is because actors enhance their own capacities by acting together (Jütting, 2020). These agreements between multiple stakeholders are seen as networks, which help to create and share new social practices (Pel et al., 2020).

Other perspectives on the ecosystem focus on geographical space, which means that national, regional, and local innovation systems exist (Edquist, 2011). However, this perspective may raise concerns about the strong diversity of rules, norms, and practices, as recognizes that comparison are difficult (Edquist, 2011, 37). Scholars such as Terstriep et al., (2020) applied a regional perspective to social innovation ecosystems, including actors, institutions, knowledge, and innovation pathways as main elements of analysis. This perspective has the advantage of showing multilayers that define each process of the innovation.

In order “to understand the ecosystem as the comprehensive organizational, institutional, and cultural setting in which the SI [social innovation] is embedded” (Kaletka et al., 2017, 85), the SISCODE cases were examined alongside a multilayered heuristic model in an explorative research process. Building upon a theoretical approach from media science, known as the “Onion-Model” (Weischenberg, 1990, 53), which strives to explain different spheres affecting journalistic acting and content generation, the heuristic provides a kind of searchlight to the right questions to ask, depending upon the research interest. The model is providing a starting point taken up and extensively adapted for social innovation research. It elaborates four units to observe: a context of norms, a context of structures, a context of functions, and a context of roles. These layers and their interrelation can be used as a lens to describe certain dynamics within a social innovation initiative or to identify and further examine drivers and barriers affecting its development:

- The **context of norms** encloses a perspective on “societal framework conditions and challenges” like “professional and ethical standards, historical and legal conditions, codes, and other accepted social standards” (Kaletka et al., 2017, 85). Hence, this context can be seen as an approach to analyze factors on the societal macro-level.
- The **context of structures** can be understood to enclose the meso-level, taking up a rather structuralist perspective. It explicitly encloses “constraints and path dependencies because of existing institutions, economic, political, and technological imperatives.” For instance, “the setup of a city administration, restricting what can be achieved on the role and functional context, or the political orientation of the government.” (ibid.)
- Both the context of functions and the context of actors are aimed at the societal micro-perspective. For the **context of roles**, the authors suggested to look at “socio-demographic factors and roles of social innovation stakeholders and beneficiaries […]. This includes these actors’ political and social attitudes, motivations, socialization, self-concepts, image, capabilities, and skills.” (ibid.)
- The **context of functions** encloses “management procedures, business, and governance models,” “how different actors are interlinked and collaborate, how they adjust their roles in a wider network context, and how the network is governed.” (ibid.)

Of course, they cannot be distinguished incisively as their overlapping is possible. Furthermore, they are highly interrelated and dependent upon another. The context factors of relevance must be determined and put into relation during the research process. Hence, the model can be understood as one possible initial structured approach to an ecosystem in which a specific social innovation process takes place to explore specific dynamics of interest and their driving and hindering factors.

In example, Komatsu Cipriani et al., (2020) applied the model for the analysis of social innovation cases “in order to understand the ability of design to foster the development of robust ecosystems” (Komatsu Cipriani et al., 2020:1012), whereas Eckhardt et al., (2017) applied it to digital social innovation and its potential for inclusive societies. In the SISCODE project, the heuristic model was adapted to examine co-creation processes alongside the findings and theoretical groundwork provided in the first project stages (Rizzo et al., 2018; Smallman and Patel, 2018). In the following section, the adaption of the heuristic model is presented.

### Application to Co-creation Initiatives

Social innovations can be the goal and result of co-creation, for instance, political innovations, technical innovations, or service innovations. In both social innovation and co-creation research, the examination of ecosystems plays a decisive role in order to achieve a comprehensive understanding of its embeddedness. Against this background, the heuristic was adapted for the analysis of co-creation by design in the European research project SISCODE and provided a basic, open, analytical grid for the data collection in the different phases of research. For this analysis, 135 cases of co-creation from all over Europe have been collected and quantitatively evaluated (Eckhardt et al., 2019). In addition, a qualitative in-depth examination of 55 cases was carried out (Eckhardt et al., 2020a; Eckhardt et al., 2020b). As a project with a European focus and cases from all over Europe, SISCODE needed an instrument that made a context-sensitive analysis possible and that could do justice to the different environments of the single and diverse co-creation cases. In this way, the heuristic serves as a central analytical tool and grid for the research activities in SISCODE. In line, the qualitative analysis of the 55 cases was based on the content of the heuristic model, and the data were coded by means of a qualitative content analysis, based on categories that go back to the heuristic model and its four contexts (i.e., norms, structures, actors and roles, and functions).

In the first phase of research, an extensive review of existing practices and literature of co-creation in RRI and RRI policies has been set up (cf. Smallman and Patel, 2018; Deserti et al., 2019). The main results fed into the heuristic as “sensitizing concepts” to enrich the presuppositions on contextual factors of influence for the processes of co-creation. The role of design as focus became a cross-cutting theme to be observed. In example, the single layers were underpinned by the following presuppositions:

- On a normative context, political and normative frameworks had to be observed, as well as the attitudes toward co-creation by design as an accepted practice were of interest. It was an attempt to elaborate the overall culture toward collaboration in different ecosystems.
- Structurally, especially descriptive factors were thought to be of interest for the embeddedness of co-creation (e.g., socioeconomic or demographic parameters) or the technological and financial equipment of an initiative, as it became clear from initial research that resources always determine the success of co-creation.
- On a functional context, it was taken into account that “Methods and objectives of co-creation need to be explicit and carefully selected to be appropriate to the subject, context, and people,” which elucidates the importance to closely examine which tools and methods were used, how, and their evaluation by different people with different roles in the process. In addition, it became evident how structural factors (regional level and institutional level) might determine the tools and instruments.
- Last, initial research emphasized the predominant significance of the “role-context,” leading to an emphasis of this layer of participating actors and their roles (e.g., as experts or lay people, interested citizens, or scientists) in the second, empirical research phase.

**FIGURE 1:**
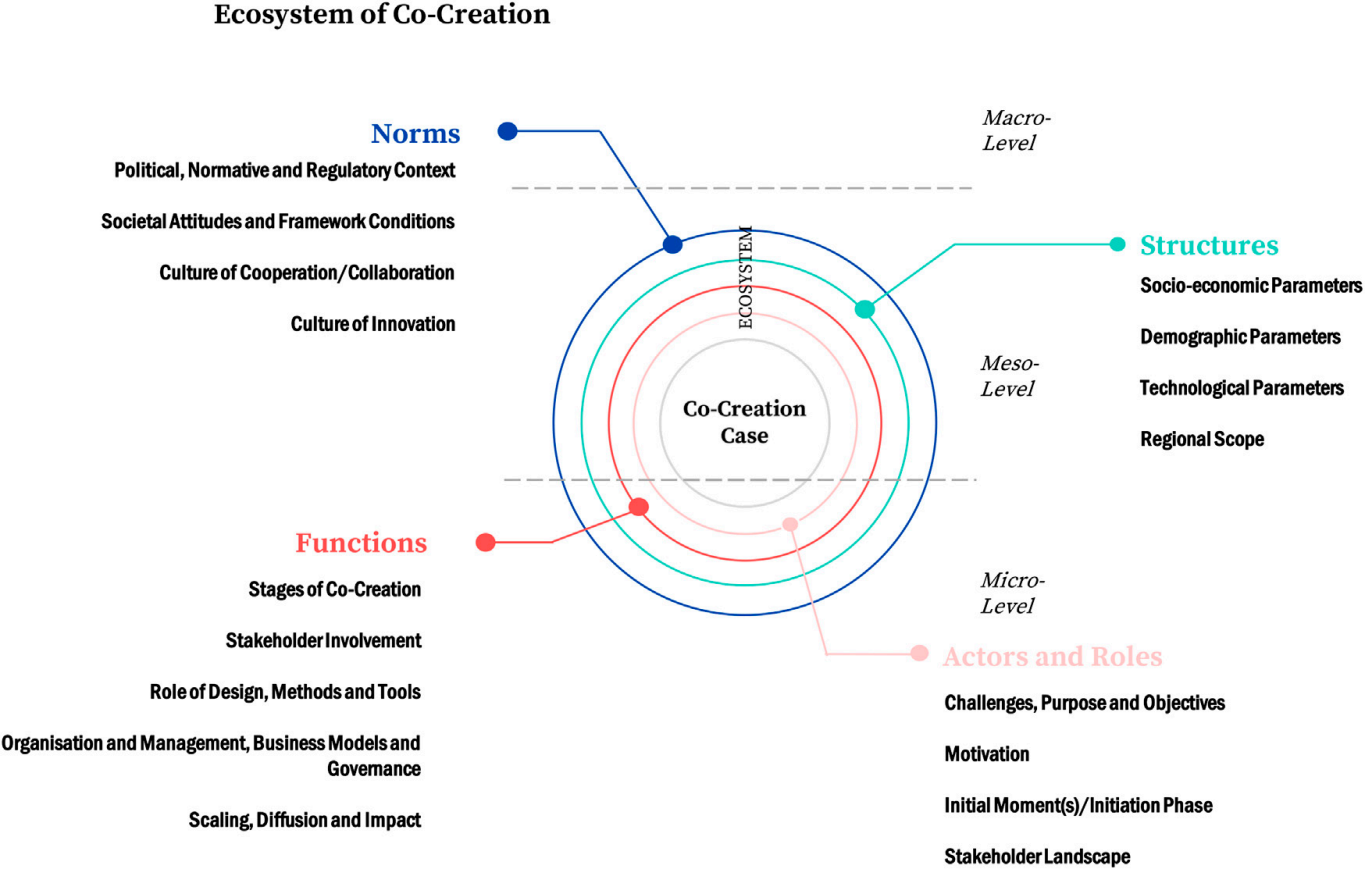
Ecosystem of Co-Creation (Source: own; based on Kaletka et al., 2017).

The adaptions resulted in the following Figure 1, as a schematic representation of the heuristic model:

To further illustrate the empirical research, the next chapter presents two examples from RRI (Ilona robot) and policy-making (Sharing City).

## Exemplary Application of the Heuristic Model

The following cases illustrate two processes of co-creation in different contexts. The two examples were selected from the collection of 55 case studies and innovation biographies from the SISCODE project (Eckhardt et al., 2020b) because they represent exemplary cases that make the different levels of the heuristic model of co-creation more visible. Thereby, these two cases were chosen because of their interesting and at some points controversial co-creation processes—Sharing City Umeå that faces more on social (new social practices through citizens engagement) than on technological innovation (of robots in elderly care) as Ilona robot. Moreover, both cases show how the heuristic model of co-creation works and which learnings arise. These cases have been further described and analyzed as innovation biographies (Iasillo, 2020; Wascher, 2020). For both cases, interviews with experts on the cases were conducted to complement information initially gained from desk research. For the first case, Ilona robot, two expert interviews were conducted, and the co-creation process was documented through the Lahti Living Lab, where researchers identify the impacts and acceptance of care robot implementation through the approach of Human Impact Assessment (Iasillo, 2020). For the second case, Sharing City Umeå, three interviews providing additional information were conducted (Wascher, 2020). The case studies and innovation biographies provide the basis for the exemplary application of the heuristic model in this chapter.

The first case, Ilona Robot in Finland (Iasillo, 2020), represents a case that introduces new technology for elderly care. This case especially shows how different stakeholders, such as municipality, researchers, and elderly care staff, worked together, and how a culture of cooperation and partnership was used in a small municipality to modernize elderly care and change perception of care services in Finland with the first robot in elderly care. The second case, Sharing City Umeå in Sweden (Wascher, 2020), shows the processes of co-creation for policy-making in sustainable cities, not only at the macro-level but also at the local level. This case especially shows the involvement of local government in the development of new solutions and partnerships with citizens and funders to manage the city's population growth through social, ecological, cultural, and economic sustainability. The following Table 1 provides an overview of the main elements of both case studies and their different layers of the heuristic model for the purpose of comparison, whereas *Ilona Robot and Sharing City Umeå* provide an exemplary analysis of striking aspects of each layer.

**TABLE 1:** Overview of the Layers of the Co-creation Ecosystem (Source: own; based on Iasillo, 2020; Wascher, 2020).

Layers of Co-Creation	Illona Robot	Sharing City Umeå
Actors	• Municipality • Researchers	• Municipality • Municipal companies
	• Elderly care staff	• Construction companies
	• Elderly	• Local government (Umeå)
	• Students of health care	• Citizens
		• Funders
Functions	• Co-creation activities to test acceptance of a robot among elderly and elderly care staff	• Co-creation to encourage participation • User-centred design study for sustainable planning
	• Participation of citizens and elderly in public health	• Users’ involvement
	• Interaction among municipality, researchers, clients (elderly), and elderly care staff	• Problem identification refers to the goal to make sustainable mobility easy and effective
		• Prototyping to develop and test new solutions
		• Idea around project is scaled with results of subprojects
Norms	• More acceptance of clients (elderly) after interactions with the new technology • Cooperation and partnership	• Sustainable urban development as a political strategy (comprehensive plan for Umeå municipality) • Partnerships
		• Part of a long-term national innovation initiative
Structures	• Demographic challenge of aging population in Finland • Elderly care services	• Promotion of climate-friendly choices in everyday life

### Ilona Robot

The case “Ilona Robot” is a design-driven phenomenon that was developed in Finland. A service robot was introduced in elderly care services in Lahti (a city in Southern Finland) in 2015–2016 to face the demographic challenge of aging population in Finnish society. Thereby, the provision of sustainable care in times of a shrinking workforce was facilitated by the interaction among ecological, economic, and social actors as well as the introduction of (new) technologies to shape the sustainable elderly care in Lahti. To do so, co-creation activities are used to introduce the humanoid care robot “Ilona” as a new technology in elderly care, considering the role of elderly patients and care professionals. This initiative comes from the Lahti municipality that started activities among city officials, researchers, and care workers in December 2015 to April 2016 to improve technology-assisted care for elderly people through robots. During the design phase, the Lahti municipal, the Lahti Living Lab, and care professionals planned co-creative activities, whereby the needs of policy-makers, researchers, and care professionals, as well as the needs of clients were considered. In the implementation phase, Ilona robot was brought into two care homes and one geriatric rehabilitation hospital chosen by the municipality. In this stage, different stakeholders participated: on the one hand, elderly as users; on the other hand, students of health care who were trained to become acquainted with new technologies in elderly care. The interaction and impact of elderly care was monitored by the Lahti Living Lab, and a change of mind was observed after seeing that clients interact with Ilona robot. Ilona robot is still in use in the three abovementioned facilities, and it is started to use in a fourth one. Overall, Ilona robot is a top-down initiative that focuses co-creation in RRI and policy-making among different stakeholders.

#### The Context of Norms

The case of the Ilona robot (Iasillo, 2020) shows the political context and the political will in the region of Lahti, where it was in the interest of the municipality to spread acceptance and familiarity with the robot for the care of the elderly. This is not only because of its will but also because the financial resources for health care are not only a matter of the central government but of different levels of government, insurance, employers, and other actors. Due to the decentralization of health care, it is possible for the regions to make more autonomous decisions and implement innovative policies in the municipalities. Besides the will of the municipalities, there is also an attitude of the Finnish society that perceives the robots as a positive element in the society (European Commission, 2015). This social attitude facilitates the introduction of innovations in health care. This case has an exemplary culture of cooperation and partnership between the municipality, the university, the public sector, and the private sector, giving place to the first robot in elderly care in the municipality.

#### The Context of Structures

As a region with a significant decline in the industry in the 1990s, Lahti has shifted from the industrial sector to the service sector. Finland, like many European countries, has a large population over 65 years old, and in the last thirty years, this population has almost doubled (13% in 1990 to 22% in 2019) (Statistics Finland's PxWeb databases, 2020). In Lahti, for example, the population over 65 years old in 2019 was above the national average of 24% (Statistics Finland's PxWeb databases, 2020). Therefore, the demography in Finland shows the need for a change in the health sector and a modernization of the elderly care. The municipality of Lahti is a region with very few universities and as such has a very low budget for research and development (R&D). Compared to Helsinki, it has a 0.9% share of R&D, while Helsinki has about 42% (Statistics Finland's PxWeb databases, 2017). The case of Ilona robot shows a structural context that promotes innovation in the municipal area due to the high levels of decentralization, but also due to the strong needs of modernization and change in elderly care.

#### The Context of Actors

The case of the Ilona robot in Lahti is interesting because of the strong involvement of local actors. For example, this initiative started with strong motivation from local residents and the municipality, which at the same time involved researchers from the Lahti University of Technology, LUT, within the framework of the Living Lab in Lahti. This cooperation aimed to integrate the main actors in elderly care, such as elderly care staff and elderly patients themselves. In addition to the participation of local residents, the municipality was very involved in this initiative. This case exemplifies a co-creative work between all the actors involved, especially between the municipality and the Living Lab researchers and between the researchers and the elderly care staff, together with the elderly patients.

#### The Context of Functions

The participation of stakeholders in the case of the Ilona robot is crucial for the implementation and acceptance of the innovation. This case also shows different stages of collaboration, such as the participation of citizens and users in the public sector. For example, the first stage was the development of an initiate from Lahti’s residents and the municipality. Second, the collaboration with the researchers from the Lahti Living Lab was a crucial space for the development and implementation of this initiative. This stage is very relevant as a space that makes policy innovation in healthcare possible, and this stage also involved healthcare students. Third, an implementation stage in which the first healthcare institutions participated in the implementation of this initiative from December 2015 to April 2016. Finally, a private company participated in the implementation by training health workers in two care homes and a geriatric rehabilitation hospital. The co-creation activities took place with the monitoring of the Lahti Living Lab in 2015 and 2016, where researchers measured the Human Impact Assessment to identify the acceptance of care robot among the elderly and elderly care staff. The interaction among elderly care staff, students of health care, and care staff took place in two care homes and one geriatric rehabilitation.

*Functions and impact*: Some of the most important stages of co-creation in this case were the sharing of responsibilities among stakeholders in the design and implementation phases. For example, during the design phase, the municipality integrated different stakeholders, which revealed the strong motivation of the public sector to collaborate, engage, and integrate the user’s perspective into the public sector. In the implementation phase, elderly patients and health workers play an important role, as they have the most interaction with the trainers from the company where the robot was purchased, as well as the interaction for the activities with the robot itself. The evaluation phase was carried out by the researchers of the Living Lab in Lahti by observing and documenting at least twenty-seven activities between Ilona's robot and the elderly patients. The impact of these activities was assessed by observing the impact of the Ilona robot on the care staff (e.g., working environment and competencies) and the impact on the clients (e.g., interaction, and physical and emotional experience).

### Sharing City Umeå

The co-creation case Sharing City Umeå (Wascher, 2020) faces the development of the city Umeå (in northern Sweden) by testing new solutions and collaborations concerning sustainability. Thereby, the project is coordinated by the local government that regards and manages the growing population of the city through social, ecological, cultural, and economic sustainability.

Based on the knowledge of a consumption habits survey in 2018 and a travel habit surveys conducted by the city years before, local stakeholders gained concrete insights into the effects different ways of traveling have on climate. In the following, new solutions concerning sustainability—especially in mobility—were tested and supported by initiatives developed by the municipality in Umeå.

In 2019, the idea of mobility service hubs brought together different types of sharing services and products to reduce peoples’ travel needs in offering alternative and sustainable mobility solutions. Therefore, from 2020 on Umeå is considered as a testing ground for service and mobility hubs to change citizens’ behavior toward sustainable mobility. To do so, six best-practice examples of service and mobility hubs in Europe were analyzed, a case study research was done, and two focus group studies were performed, whereby the first one was about general mobility of the future and the second one about sharing service and mobility solutions for the parking garage Nanna in Umeå (Eckhardt et al., 2020b, 764). In this process, it came into light that user involvement and citizens’ engagement are important to come up with feasible, sustainable solutions and to create citizens’ long-term mobility needs. “Sharing City Umeå” thus helps to promote socially sustainable development in Umeå. Furthermore, Sharing City Umeå describes co-creation that is derived from and embedded in distinct innovation systems that considers RRI in innovation strategies and funding schemes.

#### The Context of Norms

The context of norms includes a range of different factors that have a driving or hindering influence on co-creation. The case of Sharing City Umeå exemplifies how different policies can support co-creation through agenda setting on the macro-level. Sharing City Umeå is embedded in and linked to different policy programs, starting from the macro-level (Sharing Cities Sweden and Viable Cities) and down to the local level of local agendas. Furthermore, it is implemented by the municipality of Umeå, hence directly linked to its local policies toward sustainability. At the same time, Sharing City Umeå in turn consists of various subprojects. What all these different levels have in common is that they are closely related to policies aimed at achieving sustainable change. These policies thus initially offer a supporting framework for the subprojects and do not only act as starting points but also act as enablers. Of course, it has to be taken into account that the central role of such policies in the specific context of Umeå and the larger context of Sweden do also lead to path dependencies: co-creation projects that do not address the issue of achieving more sustainability may not benefit from the framework conditions enabled by policies. For the specific case of Sharing City Umeå and its subprojects as a top-down approach, however, such policies are main enablers. In addition, the policy-driven program Sharing Cities Sweden pursues and promotes a participatory approach. In this regard, this policy also represents a very specific enabler because it is fostering the establishment of an environment that is characterized by several co-creation processes in several parallel projects. In this respect, it is supportive not only for projects that may or may not contain co-creation processes but also specifically for co-creation processes themselves. Sharing Cities Sweden also strengthens the exchange between municipalities in Sweden, and this approach—at least indirectly—also strengthens the exchange between the initiators and implementers of co-creation processes across local and regional contexts. At this point, there is also a possible interaction of policy on the macro-level of the context of norms observable with the concrete design of co-creation processes on the micro-level of the context of functions.

#### The Context of Structures

The case of Sharing City Umeå is an example of how structural factors can play a role in the context of co-creation. At the same time, it shows how such structural factors can be related to norms if structures are to be changed through agenda setting and norm setting by administrative institutions. Specifically, the Municipality of Umea is planning an increase in residents by 2050 and is actively trying to design this process. This structural change in the demographic context is framed by policies that aim to improve the quality of life by strengthening sustainability. This improvement in the quality of life in the sense of sustainable change is pursued in a participatory approach in which citizens are actively involved in various subprojects of Sharing City Umeå. This interplay of the desired structural change in relation to demographics and the setting of policy agendas enables the co-creation processes that are carried out in the case of Sharing City Umeå. At the same time, the existing population structure has an effect on the realization of the co-creation processes within the subprojects on the micro-level and thus the achievement of goals on the macro-level. The population growth of Umeå is to be designed to be sustainable and citizens are to participate in the design of sustainable solutions. The case shows that this public participation actually meets with a positive response. A social environment can be assumed in which value proposition tends to be present that enables sustainable solutions with the participation of citizens. This exemplifies not only a connection between demographic structure and administrative agenda setting but also a connection between demographic structure in the sense of the composition of milieus and the success of the co-creation process insofar as stakeholders are willing to participate.

#### The Context of Actors

Diverse actors are involved in the co-creation process. On the one hand, companies focus on sustainable aspects in water, energy, or other environmental points. On the other hand, the parking space provides with the parking garage in Nanna, where the emphasis was on. This case thus integrates actors who mainly deal with the societal challenge of environment protection and sustainability. Moreover, concerning the roles of the abovementioned actors, Sharing City Umeå is quite interesting because of the actors’ overlapping roles. For example, the funder/investor motivated and supported the initiation of the initiative, which is why the role of funder/investor and the role of initiator overlapped. Another meaningful point is user involvement and citizen engagement. By involving different groups of inhabitants of Umeå, different user perspectives are considered. Moreover, all citizens had an intrinsic motivation and interest in participating. This exemplifies a co-creation process that grounds on highly motivated citizens and their willingness to participate but not to initiate co-creation processes.

#### The Context of Functions

The role of methods in the case of Sharing City Umeå is very interesting and illustrates how methods are used to select target groups as well as to collaborate. At first, the method of the stakeholder mapping was used in a workshop to explore possible target groups. After ranking, a consultancy and agency named “Hello Future” was commissioned to design a focus group study. This organization is specialized in digital transformation and facilitates services of design and innovation processes. Moreover, it creates long-term change and innovation. Due to its commission by the municipality, Hello Future designed the abovementioned focus group study with the three selected target groups (young people, families with children, and older couples without children at home), whereby a focused group was running in each target group. All are facilitated by Hello Future and started with an introduction to discuss the upcoming ideas. Thereby, the workshop leader explains and exemplifies the ideas. Hello Future recorded, took notes, and identified existing needs through the discussion. Based on the outcome, Hello Future then collected recommendations linked to user-centered and design-driven approaches. In the focus group, the participants explore their needs and thoughts in a discussion to bring in their perspective as well as to create a good understanding of their needs. Because of this selective way of participants, who were on top recruited from a Facebook campaign, this method had an impact on the projects’ results. Moreover, such focus groups are participatory workshops to exchange experiences and to discuss about sustainability in Umeå. A user-centered approach like this also brings users’ perspectives into the co-creation process and gets it forward. However, citizens have not participated in the beginning (in the designing). Thus, the focus groups frame co-creation in practice and enable an environment of collaboration. In summary, this shows that methods have an impact on co-creative activities and the way of collaboration.

## Discussion

In this article, the concept of social innovation ecosystems was applied to the field of co-creation. We argue that the ecosystemic conditions which support or hinder the successful implementation of co-creation must be carefully identified and examined in order to fully exploit co-creation as a fruitful way to tackle a challenge. To systematize the research design, these supporting and hindering factors can be assigned to different layers of such ecosystems. This article presents the application of a heuristic model of four layers and describes two examples from a comprehensive empirical analysis conducted in the European research project SISCODE. Sharing City Umeå and Ilona Robot are two cases selected from a set of 55 initiatives and co-creation processes which have contributed to the reflections and results presented.

The heuristic provides tools to identify and observe four different, yet interlinked layers: norms, structures, functions, and roles. Thereby it has to be noted that these layers rather provide an overview of the qualitative data from the case studies and biographies. Moreover, one of the main contributions of this article is the adaptation of the social innovation ecosystem model to identify the actors, their roles, and their conditions and interactions in a specific environment. For this understanding, the study of co-creation processes was of great value in identifying more precisely how co-creation is set up within a process of social innovation and how the elements of collaboration and cooperation work. In this sense, already De Vasconcelos Gomes et al., (2018) recognized the value of co-creation within ecosystems of innovation, while other authors (Kumari et al., 2019; Jütting, 2020; Pel et al., 2020) recognized the relevance of agreements and the involvement of a variety of actors in co-creation practices.

The main theoretical implication of the work presented here is that there needs to be a stronger mutual reflection and acknowledgment of theoretical contributions in the fields of social innovation and co-creation. While social innovation research will then be able to dive deeper into the potential and pitfalls of collaborative development processes, co-creation approaches can learn from SI’s perspective on (social) impact and societal transformation. In this regard, further research could delve deeper into the relationship between co-creation and social innovation. In this article, a strong proximity of both social phenomena was presented, but at the same time, the differences were highlighted. Accordingly, it seems to be promising to analyze and understand both phenomena in their common context.

One of the main limitations of this study is that it focuses on two case studies to explain a complex model of co-creation. It certainly does not provide a complete overview of all types of co-creation processes nor can it be a generalization within all social innovation processes. But the two cases analyzed in this study provide examples where all layers of an ecosystem are possible to observe and were documented. Nevertheless, we suggest that more research is needed, especially to identify the drivers and barriers of co-creation practices and their forms of institutionalization. The comparisons of two SISCODE cases are an exemplary application of the model. Both are co-creation cases because of their collaborative phases. We see that a normative setting which enables regions to make relatively autonomous decisions and implement innovative policies based on their specific challenges, in this case within the health sector, can help to motivate different actor groups to become involved in finding solutions. In the other case, the common perception of residential structures as dissatisfying helped to define a normative framework to increase the number of residents by 2050 on the one hand triggered the definition of a set of methods and tools for collaboration, and brought together local stakeholders in different roles.

Both cases exemplify how structural factors can play a role in co-creative practices and in the promotion of innovation. Especially the Sharing City Umeå case shows how complex relationships in co-creation ecosystems are. Even if the relationships are complex, relationships support co-creation—as policies in Sharing City Umeå or partnerships between the municipality in Ilona Robot. Moreover, co-creation does ground not only on relationships but also on the integration of multiple actors. This is the reason why co-creative work between all actors involved, for example, municipality, researchers, citizens, and external stakeholders, turned out as fruitful.

Co-creation is a diversified and context-dependent phenomenon. Still, the question remains whether factors can be empirically identified which are universal characteristics of co-creation and independent from particular contexts. The heuristic model could benefit from such anchor points without losing its suitability for various purposes. In contrast to such static anchor points, the model also provides a basis for better understanding dynamics unfolding throughout the co-creation’s biography. It helps to answer questions such as the following: How are co-creation practices sustained over time? In particular, what is the impact of the case on the normative layer, on the legal framework? How do societal expectations and attitudes change toward the engagement of citizens and stakeholders throughout the process of innovation or policy-making? And what opportunities and constraints in policy design can be identified which help or obstruct the development of innovation systems based on co-creation?

In sum, the application of the model to the field of co-creation is valuable both from a scientific perspective and for practitioners. Socio-scientific innovation research is interested in better understanding why initiatives succeed, why they fail, and how they contribute to distinct changes or wider transformations in society. Here, the model presented helps to identify drivers and barriers, and thereby elements of success and failure. At least in parts, it also allows to better understand transformation processes related to the initiatives, for example, when initiatives successfully work on changing the societal expectation from or attitude toward participatory policy-making in a city or region. From a very practical point of view, and this is the main practical implication here, the heuristic can serve as a “guiding light” and help to understand what works in co-creation and what not. This would require a translation of the model for practitioners’ contexts, an introduction of guiding questions to be answered, and a reproducible way to interpret the results.

In a normative sense, establishing a setting in which co-creation is made easy and becomes a routine can be considered a key factor for thriving social innovation as well as co-creation initiatives. This seems also true for major transformational projects, as “concepts like ‘smart’ or ‘green’ city can only unfold their ‘true’ value for social innovation, when they involve participative modes of governance, social, economic and technical innovation” (Terstriep et al., 2020, 896). So, the model is instructive for the design of an innovation system, especially if this is based on a comprehensive understanding of innovation which includes not only for technological but also for social innovation.
